# The silkrose of *Bombyx mori* effectively prevents vibriosis in penaeid prawns via the activation of innate immunity

**DOI:** 10.1038/s41598-018-27241-3

**Published:** 2018-06-11

**Authors:** Muhammad Fariz Zahir Ali, Indri Afriani Yasin, Takashi Ohta, Atsushi Hashizume, Atsushi Ido, Takayuki Takahashi, Chiemi Miura, Takeshi Miura

**Affiliations:** 1 Graduate School of Agriculture, Ehime University, 3-5-7, Tarumi, Matsuyama, Ehime 790-8566 Japan; 2 South Ehime Fisheries Research Center, Ehime University, 1289-1, Funakoshi, Ainan, Ehime 798-4292 Japan; 3 Department of Global Environment Studies, Faculty of Environmental Studies, Hiroshima Institute of Technology, 2-1-1 Miyake, Saeki-ku, Hiroshima, 731-5193 Japan

## Abstract

We previously identified novel bioactive polysaccharides from *Bactrocera cucurbitae* and *Antheraea yamamai* that activate innate immunity in RAW264 murine macrophages. However, in terms of potential applications in the cultivation of prawns, there were problems with the availability of these insects. However, we have now identified a polysaccharide from *Bombyx mori* that activates innate immunity in RAW264 cells and penaeid prawns. This purified polysaccharide, termed silkrose of *B. mori* (silkrose-BM), has a molecular weight of 1,150,000 and produces a single symmetrical peak on HPLC. Eight of nine constitutive monosaccharides of silkrose-BM are concomitant with dipterose of *B. cucurbitae* (dipterose-BC) and silkrose of *A. yamamai* (silkrose-AY). The major differences are found in the molar ratios of the monosaccharides. Silkrose-BM is approximately 500-fold less potent than silkrose-AY (EC_50_: 2.5 and 0.0043 μg/mL, respectively) in a nitrite oxide (NO) production assay using RAW264 cells. However, the maximum NO production for silkrose-BM and AY were comparable and higher than that of the lipopolysaccharide of *Escherichia coli*. The survival of penaeid prawns (*Litopenaeus vannamei* and *Marsupenaeus japonicus*) after infection with *Vibrio penaecida* was significantly improved by both dietary silkrose-BM and *B. mori* pupae. This suggests that silkrose-BM effectively prevents vibriosis in penaeid prawns via the activation of innate immunity.

## Introduction

Insects remain a largely unused natural resource with great potential as they are a viable and attractive source of bioactive substances^1–3^ and a sustainable source of both human food, and terrestrial and aquatic animal feed^4–7^. Recently, we identified novel bioactive polysaccharides from the pupae of the melon fly (*Bactrocera cucurbitae*) and Japanese oak silkmoth (*Antheraea yamamai*)^8,9^. These acidic polysaccharides, termed dipterose and silkrose, comprise nine monosaccharides and activate nitric oxide (NO) production and the expression of proinflammatory cytokines and interferon β via toll-like receptor 4 (TLR4)/nuclear factor-κB (NF-κB) pathway in RAW264 murine macrophages^8,9^

Prawns are one of the most traded aquaculture products in terms of commercial value^10^. Prawn farming is a rapidly growing aquaculture sector with global production reaching 7,351,350 tons in 2015^11^. However, prawn production and price fluctuations are associated with the outbreak of microbial diseases^10^. Vibriosis is one of the most common worldwide diseases in shellfish and finfish. The genus *Vibrio* comprises gram-negative curved rod bacteria that form normal bacterial flora in aquatic environments such as coastal waters and estuaries. Virulent *Vibrio* spp. strains have been responsible for severe economic losses by causing mass mortalities among penaeid prawns including the black tiger prawn (*Penaeus monodon*), Japanese tiger prawn (*Marsupenaeus japonicus*) and white shrimp (*Litopenaeus vannamei*)^12–16^. Notably, *Vibrio parahaemolyticus* is the pathogen responsible for the recent outbreak of early mortality/acute hepatopancreatic necrosis disease (EMS/AHPND) in cultured penaeid prawns first in China (2009) and then in Vietnam (2010), Malaysia (2011), Thailand (2012) and Mexico (2013)^16^. In Japan, vibriosis was prevalent from late 1980s to early 1990s when the cultivation of *M. japonicus* was intensive^17^. Despite efforts to improve culture systems and disease control, vibriosis is still evident in Japan at the present time and effective methods of prevention have been limited. The traditional vaccination techniques used in vertebrates are not considered to be applicable to crustacean species due to their lack of B and T lymphocytes^18–20^. However, the existence of specific immune memory (also termed specific immune priming) in innate immune cells such as hemocytes has recently been suggested in Arthropods^19,20^. Moreover, vaccination with formalin-inactivated white spot syndrome virus (WSSV) or its envelopes (rVP26, rVP28) has been reported to improve survival of penaeid prawns^21,22^. In this situation, bioactive substances such as silkrose and dipterose which can activate the innate immune systems of shellfish would be predicted to be effective in protecting against vibriosis.

Assuming that they could be practically applied in aquaculture however, *B. cucurbitae* and *A. yamamai* have availability issues. *B. cucurbitae* is harmful to agricultural crops and was removed from southwest islands of Japan using a sterile insect technique^23^. *A. yamamai* silk is a local and traditional industry in Japan but its production is very limited. However, *Bombyx mori* is a domesticated species of silkmoth that is taxonomically classified as a member of *Bombycoidea* superfamily, as is *A. yamamai*^24^, and is farmed worldwide to obtain silk from its cocoon. Global silk production has continued to increase and reached 192,692 metric tons in 2016^25^. This prompted us to explore the potential of *B. mori* pupae for disease protection in prawn farming. In our present study, we describe a novel bioactive polysaccharide of *B. mori* pupae that can protect penaeid prawns from vibriosis.

## Results

### Isolation of a bioactive polysaccharide from *Bombyx mori* pupae

**Figure 1:**
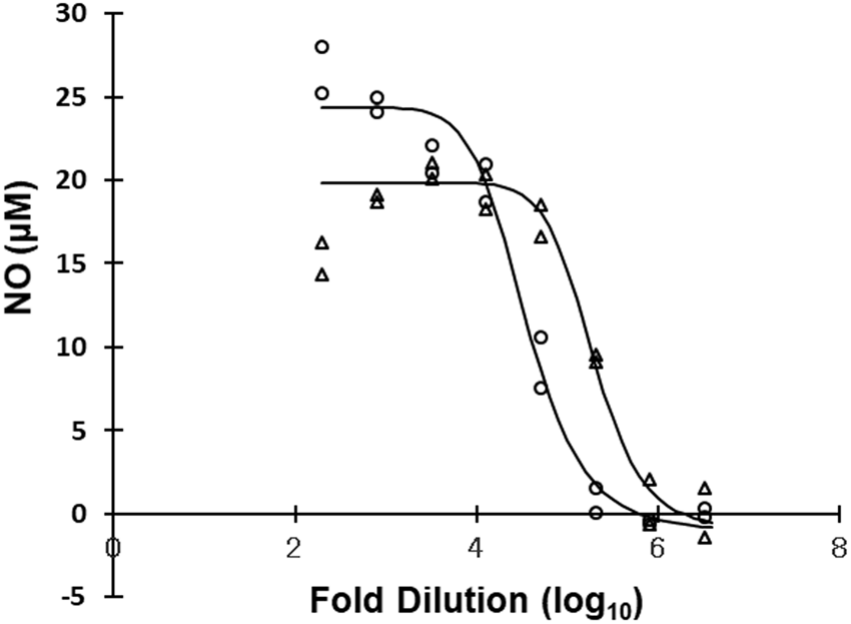
NO production activities stimulated by the addition of *B. mori* (circle) and *A. yamamai* (triangle) pupae to the culture medium of RAW264 cells.

To confirm the potency of *B. mori* pupae for innate immune activation, we first tested the NO production activity levels in RAW264 murine macrophages following the addition of crude extracts from the dried pupae. The dose response of *B. mori* pupae was agonistic in the same manner as that of *A. yamamai* (Fig. 1). The 50% effective concentration (EC_50_) of *B. mori* pupae and *A. yamamai* pupae was a 34,966 and 191,474-fold dilution, respectively.

**Figure 2:**
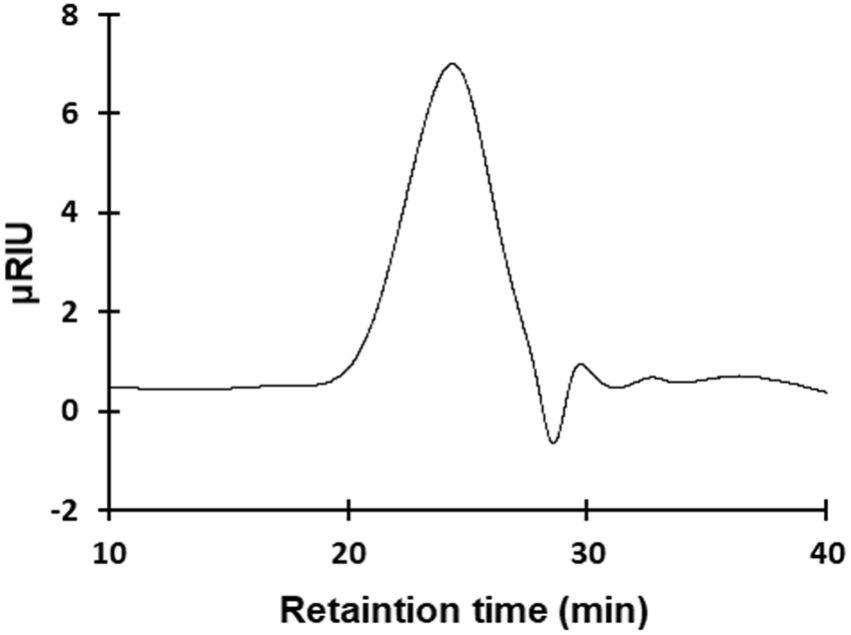
Chromatogram of the purified polysaccharide of *B. mori* pupae on size-exclusive gel filtration chromatography.

**Figure 3:**
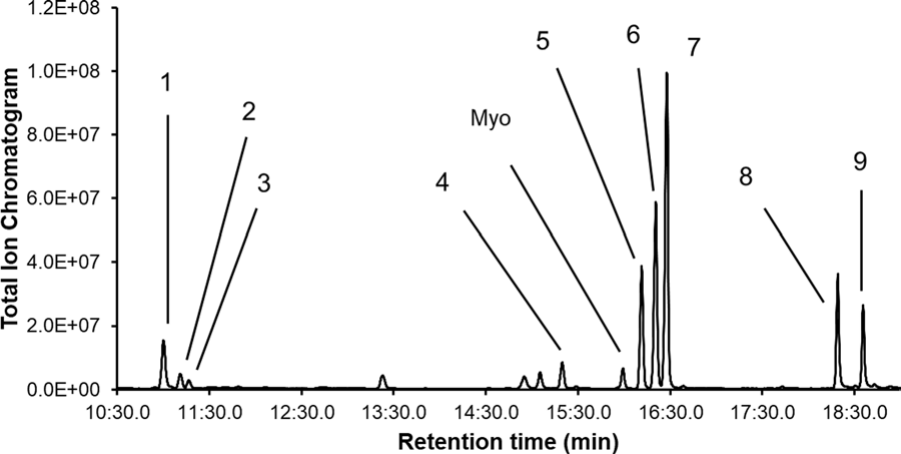
Total ion chromatogram of the purified polysaccharide of *B. mori* pupae. 1, l-rhamnose; 2, l-fucose; 3, l-arabinose; 4, d-glucuronic acid; 5, d-mannose; 6, d-glucose; 7, d-galactose; 8, N-acetyl-d-glucosamine; 9, N-acetyl-d-galactosamine. Myo, Myo-inositol, an internal standard.

After obtaining crude polysaccharides of *B. mori* pupa by water extraction and ethanol precipitation, positive fractions in the NO production assay were collected after gel filtration and anion-exchange chromatography on a fast protein liquid chromatography (FPLC) system. The purified water-soluble polysaccharide of *B. mori* was found to be a homogenous molecule that appeared as a single symmetrical peak on HPLC equipped with a size-exclusive gel filtration column (Fig. 2). The molecular weight of the purified polysaccharide was 1,150,000 as determined by HPLC using pullulans of different molecular weights (Table 1). Nine monosaccharides were identified in the purified polysaccharide of *B. mori* by GC-MS (Fig. 3) and their molar ratios are indicated in Table 2. Eight of these monosaccharides were found to be common with those among the bioactive polysaccharides of *A. yamamai* and *B. cucurbitae*, but to differ in terms of molar ratios. We termed this newly identified polysaccharide ‘silkrose of *B. mori*’ (silkrose-BM) after our previous finding of silkrose from *A. yamamai* pupae^9^.

**Table 1:** Molecular weights and EC_50_ values of the purified polysaccharides.

Species	Molecular weight^*^	EC_50_	
		μg/mL	pmol/mL
*B. mori*	1,150,000	2.5 + 0.5	2.1 + 0.4
*A. yamamai*	315,000	0.0043 + 0.0029	0.014 + 0.009
*E. coli* O26 (LPS)	N.D.	0.011 + 0.002	N.D.

^*^The molecular weight of the polysaccharide isolated from *A. yamamai* is based on Ohta *et al*.^8,9^.

**Table 2:** Molar ratios of monosaccharides in the purified polysaccharides.

Monosaccharide	Molar ratio		
	*Silkrose-BM*	*Silkrose-AY* ^*^	Dipterose-BC^*^
l-rhamnose	6.7	6.2	21.2
l-fucose	2.2	2.1	1.4
l-arabinose	0.5		
d-mannuronic acid		3.5	
d-glucuronic acid	7.5	3.5	6.2
d-mannose	9.4	9.3	8.0
d-glucose	14.6	13.1	18.2
d-galactose	23.8	48.9	16.6
N-acetyl-d-glucosamine	19.6	5.7	19.6
N-acetyl-d-galactosamine	19.6	7.6	8.5
d-ribose			0.3

^*^Based on Ohta *et al*.^8,9^.

**Figure 4:**
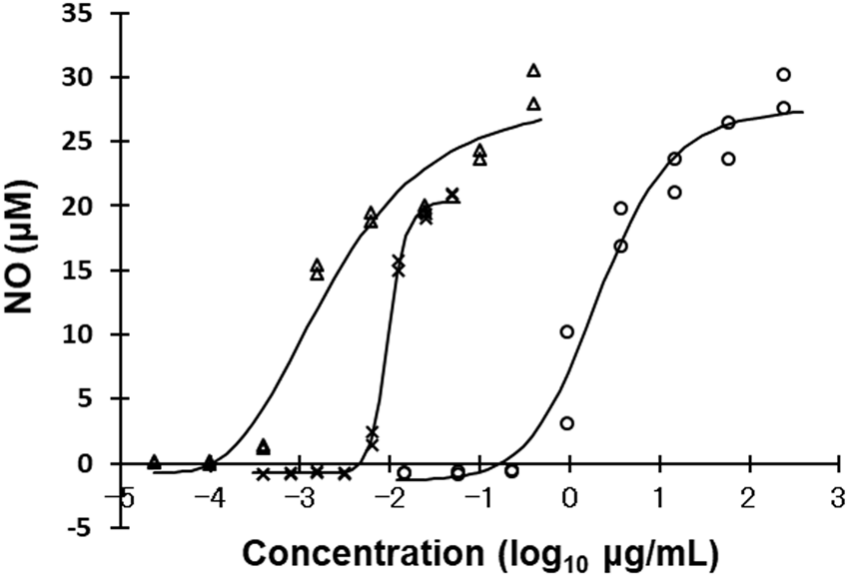
NO production activities in RAW264 cells stimulated by the polysaccharides purified from *B. mori* (circle) and *A. yamamai* (triangle) pupae. LPS of *E. coli* O26 was used as a positive control (cross).

The dose reaction of silkrose-BM was agonistic in a similar manner to silkrose of *A. yamamai* (silkrose-AY) and lipopolysaccharide (LPS) of *Escherichia coli* O26 in an NO production assay using RAW264 cells (Fig. 4). Notably, the maximum NO production values with silkrose-BM and AY were similar and higher than that of LPS. The EC_50_ of silkrose-BM, AY and LPS was 2.5 μg/mL, 0.0043 μg/mL and 0.011 μg/mL, respectively (Table 1).

### Efficacy of silkrose-BM against vibriosis in penaeid prawns

**Figure 5:**
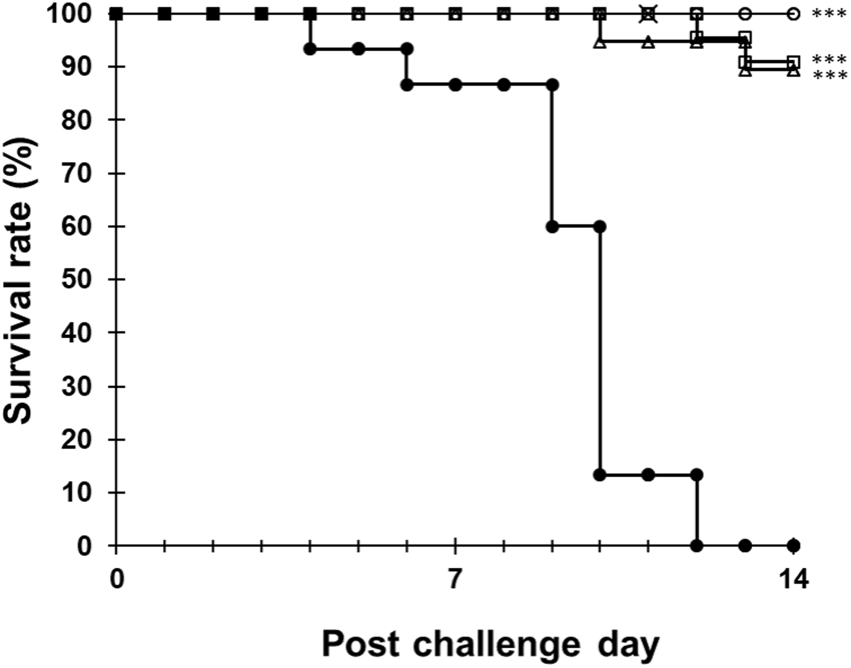
Survival curves of *L. vannamei* after immersion with the *Vibrio penaedia* IAYKG13-1 strain (input dose: 3.8 × 10^5^ cells/L). Control diet group (filled circle), 0.0125 μg/g silkrose-BM diet group (open square), 0.250 μg/g silkrose-BM diet group (open triangle) and 5 μg/g silkrose-BM diet group (open circle). The cross indicates drop-out cases due to accidental death. Asterisks indicate statistically significant differences compared with the control group by log rank test with Bonferroni correction (*p* < 0.05). *p < 0.01, **p < 0.001, ***p < 0.0001.

To confirm the efficacy of silkrose-BM against bacterial disease in penaeid prawns, *L. vannamei* was fed with dietary silkrose-BM (0, 0.0125, 0.25, 5 μg/g) for 1 month, and then infected with a virulent strain of *Vibrio penaecida* (IAYKG13-1 strain) by immersion. Dietary silkrose-BM did not cause a dose-dependent increase or decrease in prawn growth (Table S1). After infection with the challenge strain, prawn survival was significantly improved in the silkrose-BM groups compared to the control diet group (Fig. 5). On post-challenge day 14, survival rates excluding accidental deaths were 0% (0/15 prawns) in the control diet group, and 90.1% (20/22 prawns), 89.5% (17/19 prawns) and 100% (13/13 prawns) in the 0.0125, 0.25 and 5 μg/g silkrose-BM groups, respectively. Two prawns in the 5 μg/g silkrose-BM group died accidentally by jumping out of the tank.

**Figure 6:**
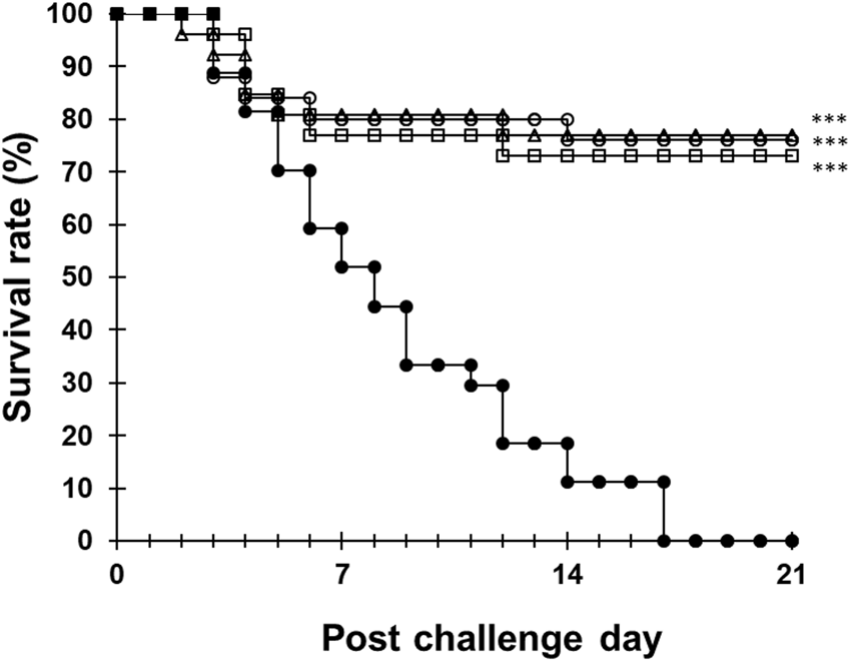
Survival curves of *M. japonicus* after immersion with the *Vibrio penaedia* IAYKG13-1 strain (input dose: 3.6 × 10^8^ cells/L). Control diet group (filled circle), 0.001% *B. mori* pupae diet group (open square), 0.01% *B. mori* pupae diet group (open triangle), 0.1% *B. mori* pupae diet group (open circle). Asterisks indicate statistically significant differences compared with the control group by log rank test with Bonferroni correction (*p* < 0.05). *p < 0.01, **p < 0.001, ***p < 0.0001.

For potential future field use, we further confirmed the efficacy of *B. mori* pupae containing silkrose-BM. *M. japonicus* was fed with experimental diets for 2 weeks and then challenged with *Vibrio penaecida* by immersion. *B. mori* pupae did not cause a dose-dependent decrease of the growth of prawns over a feeding period of 2 weeks, but instead showed a tendency towards increased growth (Table S2). After infection, prawn survival was significantly improved in the *B. mori* pupae diet groups compared to the control diet group (Fig. 6). The survival rates on post-challenge day 21 were 0% (0/27 prawns) in the control diet group, and 73.1% (19/26 prawns), 76.9% (20/26 prawns) and 76.0% (19/25 prawns) in the 0.01%, 0.01% and 0.1% *B. mori* pupae diet groups, respectively.

## Discussion

The widely accepted concept of innate immunity is that its activation is initiated by the recognition of foreign substances, known as pathogen associated molecular patterns (PAMPs), by pathogen recognition receptors (PRRs)^26^. In our current study, we identified a bioactive polysaccharide from *B. mori* pupae that can activate innate immunity in murine RAW264 cells. Moreover, dietary supplementation of silkrose-BM resulted in an improved survival of penaeid prawns after their infection with a virulent *Vibrio* strain. Our findings thus suggest that silkrose-BM serves as a PAMP for both mammals and crustaceans. In general, PAMP-containing polysaccharides affect a wide range of animal cells and species including vertebrates and invertebrates, as reported for LPS and β-glucans^27–36^.

It is notable in our current analyses that crude *B. mori* pupae were effective against vibriosis in *M. japonicus*, which is a representative penaeid prawn in commercial aquaculture. We cannot however exclude the possibility that other substances contained in the *B. mori* pupae such as anti-microbial peptides^37–39^ and chitin^40^ play a role in this anti-bacterial protection together with silkrose-BM, or that uncharacterized inhibitory molecules might interfere with the action of silkrose-BM. However, our findings also indicate that *B. mori* pupae can be used without additional purification. In addition to their availability as a by-product of silk production, the ease of processing of these pupae would be highly advantageous for their practical application in aquaculture.

We employed the NO production assay using RAW264 cells in our present study to monitor innate immune activation for the reasons outlined below. The promoter of the murine gene encoding inducible nitric oxide synthase (iNOS) contains NF-κB binding sites and the transcriptional induction of the iNOs gene is strictly dependent on the activation of the NF-κB signaling pathway^27^. Furthermore, we have confirmed in previous studies that the NO production activities promoted by dipterose and silkrose are tightly associated with innate immune responses in RAW264 cells^8,9^.

The bioactive polysaccharides that we have identified from insects are characterized by constitutive monosaccharides. Eight of nine monosaccharides are common among silkrose-BM and AY and dipterose of *B. cucurbitae* (dipterose-BC). The monosaccharides that differ among these species are l-arabinose, d-mannuronic acid and d-ribose but the molar ratios of these molecules are small. Hence all, or at least a significant part, of the eight shared monosaccharides will likely participate in the formation of recognition sites for PRRs. Clear differences in the polysaccharides of silkrose-BM and AY and dipterose-BC are found in the molar ratios of the monosaccharides and molecular weights that likely explains differences in the potency, monosaccharide alignment and three-dimensional structure of the polysaccharides.

We did not show the cellular and molecular recognition mechanism of silkrose-BM in our current study. However, the dose response of silkrose-BM in the NO production assay is typically agonistic in the same manner as that of LPS of *E. coli* O26 and silkrose-AY, and is consistent with our previous observations^8,9^. Furthermore, the constitutive monosaccharides of silkrose-BM are closely similar to those of silkrose-AY and dipterose-BC, suggesting similar cellular recognition mechanisms between these bioactive polysaccharides.

One reported mechanism that underlies the functions of insect bioactive polysaccharides in RAW264 cells is the TLR4/NF-κB pathway^8,9^. Among the well-characterized bioactive molecules containing polysaccharides, bacterial LPS is also known to activate the innate immune system in mammalian cells via TLR4^36^. Thus, the cumulative evidence on the activities and functions of LPS, rather than the β-glucans which consist of glucose polymers forming a triple helical structure and are recognized via cell surface receptors such as CR3, lactosylceramide, scavenger receptors and dectin-1 in vertebrates^28,30^, provides some clues to the cellular recognition mechanisms of silkrose and dipterose in mammalian cells. During the activation of TLR4 by LPS, homo- and heterotypic multiple receptor complexes, that incorporate CD14, MD2 and TLR4 as core molecules, form an activation cluster^36^. We speculate that participants in the receptor complex for the recognition of insect polysaccharides do not entirely overlap with those for bacterial LPS due to the lack of lipid A in the insect polysaccharides and differences in the constitutive monosaccharides.

In higher invertebrates, genetic studies in *Drosophila* have revealed that three distinct signaling pathways via Toll, Immune Deficiency (IMD) and Signal Transducer and Activator of Transcription (JAK/STAT) are crucial for the immune response to microbial infection^41^. An RNAi knockdown of the *L. vannamei Toll* gene (*LvToll*) increases mortality and reduces bacterial clearance after challenge with *Vibrio harveyi*^42^. Furthermore, the expression of *LvToll* and a Chinese prawn, *Fenneropenaeus chinensis Toll* (*FcToll*) gene are modulated after *Vibrio harveyi* and *Vibrio anguillarum* infection, respectively^42,43^. These findings suggest the possibility that the Toll pathway participates in the innate immune activation and protection from vibriosis by the bioactive polysaccharides of insects. However, prawns have an insect-type Toll^43–46^ which does not directly serve as a receptor for foreign substances^41,47^. Other humoral, cell surface and intracellular molecules have been identified as PRRs in higher invertebrates^41,47,48^. To date, 10 distinct PRR families are known in prawns^48^. The elucidation of PRRs and their downstream responses will be crucial to our fuller understanding of the innate immune pathways in prawns that are activated by the bioactive polysaccharides of insects.

Protection from infectious disease during prawn cultivation has been mainly achieved by the generation of specific pathogen-free (SPF) stocks^49,50^, improvements to culture systems and public control of infectious diseases. However, *Vibrio* spp. form normal flora in the ponds where prawns are cultured. Furthermore, bacteria can acquire virulent genes through horizontal gene transfer caused by naked DNA uptake, bacteriophage infection and bacterial conjugation between homogenous and heterogenous species^51–53^. Hence it is difficult to absolutely eliminate pathogenic bacteria from fish farms. Antimicrobial agents are effective against bacterial disease, but their usage is restricted to minimize impacts to the natural environment and prevent the generation of antibiotic resistant strains. In contrast, bioactive substances that function through innate immunity are free from such concerns. In conclusion, bioactive substances like silkrose will become more important tools for bacterial disease protection in future prawn cultivation.

## Methods

### Purification of bioactive polysaccharides

Insect-derived bioactive polysaccharides were purified as described previously^8,9^. Briefly, crude polysaccharides obtained from dried powdered *B. mori* pupae by water extraction and ethanol precipitation were fractionized and purified by gel filtration and anion-exchange chromatography on a fast protein liquid chromatography (FPLC) system. A HiPrep 26/60 Sephacryl S-500HR column (GE Healthcare, Chicago, IL) and HiPrep DAEA FF 16/10 column (GE Healthcare) were used for gel filtration and anion-exchange chromatography, respectively. Bioactive polysaccharides were enriched by ethanol precipitation after collecting positive fractions in an NO production assay. Purified polysaccharide concentrations were determined using the phenol-sulfuric acid method with D-glucose standards used to determine total sugar levels.

### Determination of molecular weights of bioactive polysaccharides

Molecular weights of the purified polysaccharides were determined by gel filtration chromatography using a high-performance liquid chromatography (HPLC) system as described previously^8,9^. Briefly, 1 mg/mL of purified polysaccharide dissolved in 0.2 M phosphate buffer (pH 7.5) was applied to a Showdex SB-807 HQ size-exclusion chromatographic column (Showa Denko K.K., Tokyo, Japan) after filtration with a 0.22-μm filter. The column was maintained at 35 °C. The applied samples were eluted with 0.2 M phosphate buffer (pH 7.5) at a flow rate of 0.5 mL/min and detected by a refractive index detector. Preliminary calibration of the column was conducted using pullulans of different molecular weights (pullulan P-5, P-10, P-20, P-50, P-100, P-200, P-400, P-800 and P-2500). Molecular weights were calculated from the pullulan calibration curve.

### Determination of monosaccharide compositions

Determination of the monosaccharide composition of the immunostimulatory polysaccharide was performed as described previously^8,9^. Briefly, the isolated polysaccharide (100 μg) was hydrolyzed with 2 M trifluoroacetic acid at 100 °C for 16 hours. The hydrolyzed products were then evaporated using an N_2_ stream and converted to alditol acetates by successive NaBH_4_ reduction and acetylation with Ac_2_O-pyridine (1:1, v/v) following the method described by Sassaki *et al*.^54^. GC-MS analysis was performed on a gas chromatography system equipped with HP-5 capillary column (Agilent Technologies, Santa Clara, CA) connected to a mass spectrometer. Helium was used as the carrier gas.

### NO production assay

RAW264 murine macrophages were used in the NO production assay and were obtained from a Cell Bank (Riken Bioresource Center, Tsukuba, Japan) and cultured in minimum essential medium (MEM) supplemented with 10% fetal bovine serum, 0.1 mM non-essential amino acids, 100 U/mL penicillin and 100 μg/mL streptomycin. Cells were kept at 37 °C in a 5% CO_2_ humidified atmosphere. In the assay, 10^5^ cells were plated in each well of a 96-well plate and cultured for 2.5–3 hours. A dilution series of pupa extracts or purified polysaccharides was applied to the wells which were then cultured for 24 hours at 37 °C. The nitrite concentrations in the supernatant of the cultured medium were measured using a Griess reagent kit (Promega, Madison, WI) in accordance with the manufacturer’s instructions.

### Preparation of dried pellets

The compositions of the base diets for *L. vannamei* and *M. japonicus* are listed in Tables S3 and S4, respectively. Dry ingredients were mixed well, supplemented with fish oil, and finally mixed with water before pelletization using a noodle maker HR2365/1 (Philips, Nederland). The ingredients were mixed well again when adding the oil and water. After granulation, the pellets were completely air-dried at 60 °C for 1–3 days. (note: the pellets of *M. japonicus* were steamed for 10 minutes before air-drying to promote the denature of wheat flour and gluten and thus prevent the pellets from dissolving in water when feeding.) Purified silkrose or defatted and powdered *B. mori* pupae were suspended in water and then mixed with other ingredients.

The composition of silkrose-BM in the experimental diets was 0, 0.0125, 0.25 and 5 μg/g, respectively. Powdered *B. mori pupae* were mixed into the base diets at estimated content levels of 0.874, 8.74 and 87.4 μg/g silkrose-BM in the 0.001%, 0.01%, and 0.1% *B. mori* pupae diet groups, respectively. No ingredients were replaced when adding the purified silkrose or *B. mori* pupae, since the supplemented amounts were very small (equal to or less than 5 μg/g or 0.1%) and the nutritional influence of silkrose or *B. mori* pupae was considered negligible.

### Challenge study

*L. vannamei* was kindly donated by the Oita Marine Biological Technology Center of Nissui (Oita, Japan). *M. japonicus* was a generous gift of Higashimaru Co., Ltd. (Kagoshima, Japan) and the Fisheries Research Center at the Ehime Prefectural Research Institute of Agriculture, Forestry and Fisheries (Uwajima, Japan).

Prawns fed with the experimental diets (5% for body weights/day) for 1 month (*L. vannamei*) or 2 weeks (*M. japonicus*) were used in the challenge study. In the pre-challenge phase, prawns were cultured in 250 L round tanks. A single tank was allocated to each study group. The number of enrolled prawns are indicated in Tables S1 and S2. Sand filtrated-natural seawater was supplied by a flow-through system. For the culture of *L. vannamei*, water was supplied from above the tanks and drained from the bottom of the tanks. For the culture of *M. japonicus*, the tank bottom was provided with sand substrate and water was supplied from the bottom of the tanks. The water temperature was kept at 28 °C (*L. vannamei*) or 18–22 °C (*M. japonicus*).

The challenge strain (*Vibrio penaecida* IAYKG13–1) has been described previously^55^. To prepare challenge inoculums, the bacterial stock was aseptically injected into marine broth and cultured for 2 days at 28 °C with vigorous agitation. For *L. vannamei* infection, 16 mL of bacterial suspension was added to 2 L of 50% artificial sea water in which the prawns were immersed for 1 hour at 28 °C. After infection, the prawns were cultured in 50% artificial seawater at 28 °C in duplicate 10 L tanks equipped with an underwater air-filtration system. For *M. japonicus*, 40 mL of bacterial suspension was added to 5 L sea water in which the prawns were immersed for 1 hour and then cultured in duplicate 45 L tanks with a bottom filtration system. The water temperature was maintained at 18–22 °C throughout the study for *M. japonicus*.

The bacterial numbers in the challenge inoculums were counted by plating on marine broth agars. Experimental diets (3–5% for body weights/day) were used in the post-challenge periods. The challenge phases ended at 2–5 days after death of control prawns stopped.

### Data analysis

Regression curves were obtained from duplicate measurements of NO production for pupae extracts and purified polysaccharides using curve fitting to the following formula on ImageJ software^56^:

$${\rm{y}}={\rm{d}}+({\rm{a}}-{\rm{d}})/(1+{({\rm{x}}/{\rm{c}})}^{{\rm{b}}})$$

where x is the dilution of pupal extracts (log_10_ fold dilution) or concentration of silkrose-BM (log_10_ μg/mL) and y is the concentration of NO (μmol/L).

The silkrose-BM content in the pupae was estimated as follows:

$$\begin{array}{rcl}{\rm{Silkrose}}-{\rm{BM}}\,{\rm{contentin}}\,{\rm{pupae}}\,({\rm{mg}}/{\rm{g}}) & = & {{\rm{EC}}}_{50}\,{\rm{of}}\,{\rm{pupal}}\,{\rm{extracts}}\,({\rm{fold}}\,{\rm{dilution}}\,({\rm{mL}}/{\rm{g}}))\\  &  & \times \,{{\rm{EC}}}_{50}\,{\rm{of}}\,{\rm{silkrose}}\,-{\rm{BM}}\,({\rm{mg}}/{\rm{mL}})/1000\end{array}$$

In the challenge study, the Kaplan-Meier method and log rank test with a Bonferroni correction were used for the analysis of survival curves (*p* < 0.05). The Jonckheere-Terpstra test (two-tails, *p* < 0.05) and Steel Dwass multiple comparison test (*p* < 0.05) as a post hoc test were used to analyze the body weights of the prawns.

## Electronic supplementary material

Table S1, S2, S3, S4
