# Supplementary material for: The silkrose of Bombyx mori effectively prevents vibriosis in penaeid prawns via the activation of innate immunity
Source: Sci Rep. 2018 Jun 11;8:8836. doi: 10.1038/s41598-018-27241-3 (PMC5995915; doi:10.1038/s41598-018-27241-3)
Supplement: Supplementary file 1 — Table S1, S2, S3, S4 [file 41598_2018_27241_MOESM1_ESM.doc]

**Supplementary Tables**

**The silkrose of *Bombyx mori* effectively prevents vibriosis in penaeid prawns via the activation of innate immunity**

Muhammad Fariz Zahir Ali^1^, Indri Afriani Yasin^1^, Takashi Ohta^2^, Atsushi Hashizume^1^, Atsushi Ido^2^, Takayuki Takahashi^1^, Chiemi Miura^1, 3^ and Takeshi Miura^1, *^

^1^Graduate School of Agriculture, Ehime University, 3-5-7, Tarumi, Matsuyama, Ehime, 790-8566, Japan

^2^South Ehime Fisheries Research Center, Ehime University, 1289-1, Funakoshi, Ainan, Ehime, 798-4292, Japan

^3^Department of Global Environment Studies, Faculty of Environmental Studies, Hiroshima Institute of Technology, 2-1-1 Miyake, Saeki-ku, Hiroshima 731-5193, Japan

* Correspondence: Takeshi Miura (e-mail: miutake@agr.ehime-u.ac.jp)

***Table S1. Growth of Litopenaeus vannamei on diets containing purified silkrose of*** B. mori

|  |  | n |  |  |  | Dead prawns | |  | BW (g) |  |  |  | BWG (%)*** | |
| --- | --- | --- | --- | --- | --- | --- | --- | --- | --- | --- | --- | --- | --- | --- |
| Test | Dose (μg/g) | Initial | 2 wk** | 4 wk |  | Accidental | Unidentified |  | Initial | 2 wk | 4 wk |  | 2 wk | 4 wk |
| 1 | 0 | 47 | 42 (22) | 15 |  | 5 | 7 |  | 0.60+0.30 | 1.59+0.68 | 2.43+1.23 |  | 167.3 | 307.3 |
|  | 0.0125 | 47 | 44 (24) | 22 |  | 2 | 4 |  | 0.67+0.32 | 1.69+0.69 | 2.27+0.79 |  | 154.4 | 240.6 |
|  | 0.25* | 48 | 35 (15) | 14 |  | 4 | 10 |  | 0.81+0.39 | 1.42+0.75 | 1.47+0.70 |  | 75.7 | 82.3 |
|  | 5 | 48 | 35 (15) | 14 |  | 3 | 13 |  | 0.60+0.39 | 1.78+0.75 | 2.62+0.70 |  | 197.1 | 336.9 |
|  |  |  |  |  |  |  |  | *p* | 0.991 | 0.951 | 0.632 |  |  |  |
| 2 | 0 | 50 | 49 (29) | 22 |  | 3 | 5 |  | 1.03+0.44 | 1.27+0.48^a^ | 2.97+0.99 |  | 22.8 | 186.7 |
|  | 0.0125 | 50 | 46 (26) | 20 |  | 1 | 9 |  | 1.09+0.48 | 1.60+0.51^b^ | 3.84 +1.23 |  | 47.4 | 253.3 |
|  | 0.25 | 50 | 50 (30) | 21 |  | 1 | 8 |  | 1.07+0.57 | 1.31+0.60^a^ | 2.99+0.68 |  | 22.7 | 179.1 |
|  | 5 | 50 | 39 (19) | 18 |  | 4 | 8 |  | 1.12+0.47 | 1.81+0.65^b^ | 3.97+1.22 |  | 62.4 | 256.0 |
|  |  |  |  |  |  |  |  | *p* | 0.616 | 0.00927 | 0.0756 |  |  |  |

*The autofeeder had technical trouble during the holiday season in the 0.25 μg/g dietary silkrose group in test 1.

**20 prawns in each group were purposely removed at 2 weeks for use in another study. The number of prawns remaining after this removal is indicated in the brackets.

***BWG (%) = BW gain (g) / initial BW (g) × 100

Body weights (BW) are indicated by a mean + S.D. *p* values were obtained by the Jonckheere-Terpstra test (two-tails, *p* < 0.05).

**Table S2. Growth of *Marsupenaeus japonicus* on *B. mori*** pupae diets

|  |  | n |  |  | BW (g)* |  |  |
| --- | --- | --- | --- | --- | --- | --- | --- |
| Test | Pupae (%) | Initial | 2 wk |  | Initial | 2 wk | BWG (%) |
| 1 | 0 | 21 | 21 |  | 0.88+0.22 | 1.10+0.22^a^ | 24.9 |
|  | 0.001 | 21 | 21 |  | 0.81+0.17 | 1.15+0.15^ab^ | 42.4 |
|  | 0.01 | 21 | 21 |  | 0.90+0.20 | 1.31+0.21^bc^ | 44.7 |
|  | 0.1 | 20 | 20 |  | 0.99+0.43 | 1.44+0.44^c^ | 45.0 |
|  |  |  |  | *p* | 0.265 | 1.29x10^-5^ |  |
| 2 | 0 | 27 | 27 |  | 1.01+0.23 | 1.07+0.18^a^ | 5.9 |
|  | 0.001 | 26 | 25 |  | 1.10+0.21 | 1.36+0.20^b^ | 23.6 |
|  | 0.01 | 26 | 26 |  | 1.06+0.32 | 1.42+0.30^b^ | 34.2 |
|  | 0.1 | 25 | 25 |  | 0.91+0.26 | 1.35+0.35^b^ | 47.8 |
|  |  |  |  | *p* | 0.0909 | 4.64x10^-6^ |  |

*BWG (%) = BW gain (g) / initial BW (g) × 100

Body weights (BW) are indicated by a mean + S.D. Different letters indicate statistically significant differences according to the Steel Dwass multiple comparison test as a post hoc test after analysis with the Jonckheere-Terpstra test (two-tails, *p* < 0.05). Note that a single prawn died accidentally in the 0.001% dietary *B. mori* pupae group in test 2 due to jumping out of the tank.

**Table S3. Composition of the base diet for *Litopenaeus vannamei***

| Component | Ratio (w/w%) |
| --- | --- |
| Fish meal | 24.5 |
| Squid meal | 2.5 |
| Krill meal | 2.0 |
| Soya bean meal | 9.5 |
| Wheat flour | 48.4 |
| Wheat gluten | 4 |
| Soya lecithin | 2 |
| Cod fish oil | 3 |
| Cholesterol | 0.234 |
| Vitamin mix | 0.4 |
| Mineral mix | 0.06 |
| Choline chloride | 0.115 |
| Vitamin C | 0.071 |
| Potassium phosphate, dibasic | 0.56 |
| Calcium phosphate, mono basic | 0.56 |
| Sodium phosphate, dibasic | 0.56 |
| Magnesium Sulfate Hydrate | 1.5 |

**Table S4. Composition of the base diet for *Marsupenaeus japonicus***

| Component | Ratio (w/w%) |
| --- | --- |
| Fish meal | 20 |
| Squid meal | 30 |
| Krill meal | 20 |
| Wheat flour | 3 |
| Wheat gluten | 14 |
| Soya lecithin | 2 |
| Cod fish oil | 4 |
| Cholesterol | 1 |
| Vitamin mix | 1 |
| Mineral mix | 0.8 |
| Vitamin C | 0.1 |
| Calcium carbonate | 1.5 |
| Monocalcium phosphate | 0.5 |
| Carboxymethylcellulose | 2.1 |
